# Clinical and molecular description of two cases of neonatal diabetes secondary to mutations in *PDX1*

**DOI:** 10.1530/EDM-22-0383

**Published:** 2023-07-07

**Authors:** Nicolas Forero-Castro, Luis Carlos Ramirez, Juan Carlos Celis, Fernando Arturo Silva Henao, Fernando Leal Valencia

**Affiliations:** 1 Maternal and Child Unit of the Tolima Province, Colombia; 2 Hospital Militar Central, Bogotá, Colombia

**Keywords:** Paediatric, Male, Female, Hispanic or Latino - Central American or South American, Colombia, Pancreas, Diabetes, New disease or syndrome: presentations/diagnosis/management, July, 2023

## Abstract

**Summary:**

Pancreatic dysgenesis (PD) is a rare congenital disease, with less than 100 cases reported in the literature. In most cases, patients are asymptomatic and the diagnosis is made incidentally. In this report, we present the case of two brothers with a history of intrauterine growth retardation, low birth weight, hyperglycemia, and poor weight gain. The diagnosis of PD and neonatal diabetes mellitus was made by an interdisciplinary team composed of an endocrinologist, a gastroenterologist, and a geneticist. Once the diagnosis was made, treatment with an insulin pump, pancreatic enzyme replacement therapy, and supplementation with fat-soluble vitamins was decided. The use of the insulin infusion pump facilitated the outpatient treatment of both patients.

**Learning points:**

- Pancreatic dysgenesis is a relatively rare congenital anomaly; most of the time, patients are asymptomatic and are diagnosed incidentally.
- The diagnosis of pancreatic dysgenesis and neonatal diabetes mellitus should be made with an interdisciplinary team.
- Due to its flexibility, the use of an insulin infusion pump facilitated the management of these two patients.

## Background

Pancreatic dysgenesis (PD) is a clinical condition characterized by the partial or total absence of the pancreas, presenting genetic heterogeneity, and may be caused by a homozygous or compound heterozygous mutation in the *PDX1* gene. Mutations in this gene are a rare cause of dysgenesis of the pancreas, which presents with endocrine and exocrine manifestations, one of them being permanent neonatal diabetes, a condition that occurs in 1 in 400 000 births (1). PD presents with intrauterine growth retardation, little weight-to-stature progression, exocrine pancreatic insufficiency, neonatal insulin-dependent diabetes mellitus, and low or absent levels of C-peptide and glucagon. Of these manifestations, the most reported is hyperglycemia (2), and no dysmorphic phenotypes have been reported among those affected.

There are no precise data on PD prevalence. The following will be the report of two cases of PD due to a compound heterozygous mutation of the *PDX1* gene in two pediatric patients, male and female siblings, respectively, in the city of Ibagué, Colombia.

## Case presentation

## Case number 1

**Figure 1:**
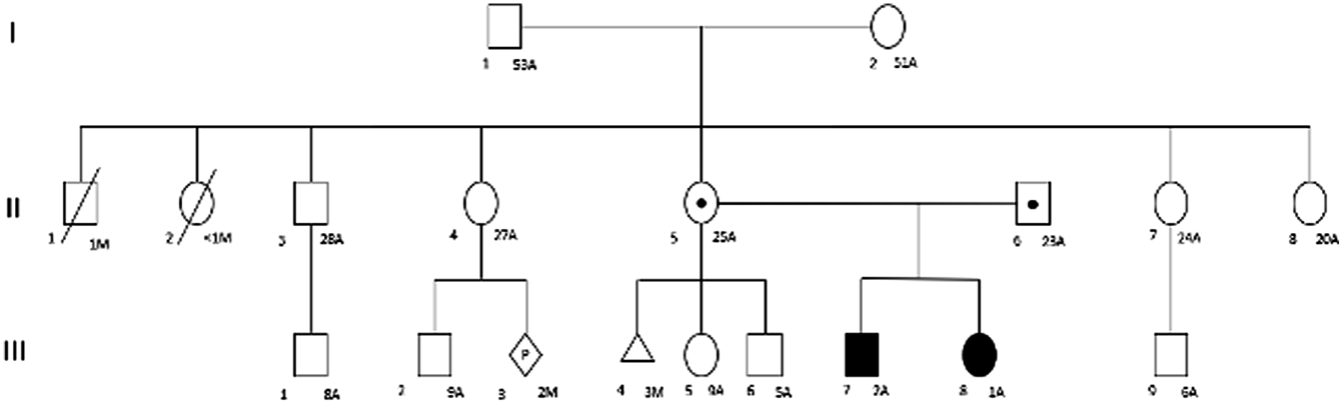
Genealogical tree: A (years) M (months). The squares represent men; circles represent women. The colored figures indicate the cases of PA. Figures with black dots indicate obligate carriers of the variants in the PDX1 gene. Figures with diagonal lines indicate death. The diamond indicates undetermined sex, and the triangle indicates gestational loss.

A 3-year-old male patient, a product of the fourth pregnancy and the couple’s first child (Fig. 1), with no history of consanguinity, with a personal history of intrauterine growth restriction. Labor occurred at 35 weeks of gestation. The weight at birth was 1440 g and the height was 45 cm. He was admitted to the Unidad Materno Infantil del Tolima (UMIT) of the department of Caquetá, Colombia, at approximately 3.5 months of age with a weight of 1805 g, height of 45 cm, HC of 32 cm, and blood glucose level of 246 mg/dL.

**Figure 2:**
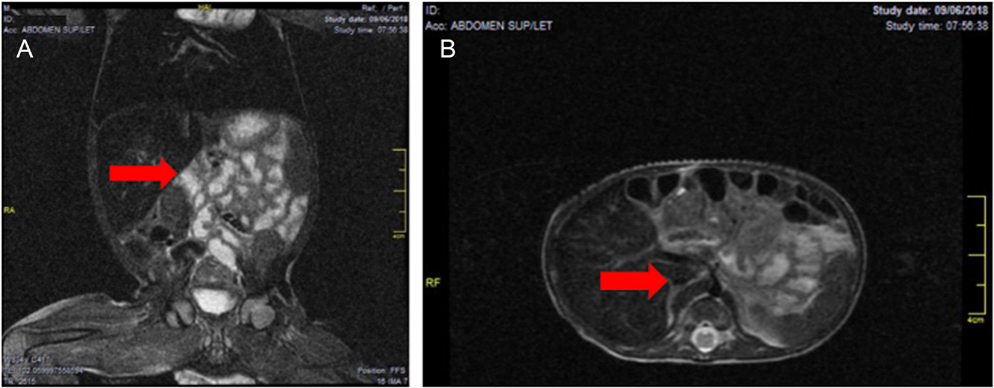
Nuclear magnetic resonance images of the abdomen of the first male patient case: (A) coronal section and (B) sagittal section; the arrow indicates the site where the pancreas should be visualized.

The cause of remission was hyperglycemia with little weight gain. Before admission to the UMIT, he was managed with insulin in continuous infusion and later with subcutaneous insulin, and from 2 months, they began management with glibenclamide 13/mg/k/day and insulin. Table 1 presents the results of the blood tests at the time of admission to the UMIT, of which the C-peptide value (0.05 ng/mL) stood out. Given the characteristics of the patient, complementary images were requested; the total abdominal ultrasound showed PD, for which an abdominal nuclear magnetic resonance was indicated, which confirmed the total absence of pancreatic tissue (Fig. 2). The genetics team suggested carrying out a molecular study by sequencing the *PDX1* and *PTF1A* genes, related to PD.

**Table 1:** Blood analysis of patient cases 1 and 2. The baseline values for the two cases are presented. Case 1 was 3 and half months old and Case 2 was 3 months old at the time of reporting these values.

Parameters	Case 1	Case 2
Leucocytes, /mm^3^	12 200	8600
Neutrophils, %	23	25
Lymphocytes, %	72	70
Hemoglobin, g/dL	12.3	12.9
Hematocrit, %	36.1	38.6
HbA1C, %	10.4	8.1
PLT, /mm^3^	551 000	203 000
BUN, mg/dL	9.2	9.9
Creatinine, mg/dL	1.2	1.0
Glucose, mg/dL	225	216
TGO, U/L	83	45
TGP, U/L	79	48
Albumin, g/dL	3.3	No data
Total Proteins, g/dL	5.2	No data
Free T4 , pmol/mL	11.3	12.8
C-peptide, ng/mL	0.05	0.05
Fecal elastase^†^, µg/g	5.2	5.0

^†^Normal value: higher than 20 µg/g.

## Case number 2

A 2-year-old female patient, a product of the fifth pregnancy but the couple’s second child, born 14 months after patient case number 1 (Fig. 1), with a history of intrauterine growth restriction, born at the 30th week of gestation, birth weight of 1090 g, height of 31 cm, and head circumference (HC) of 32 cm. The patient was also referred from the department of Caquetá at 3 months of age due to hyperglycemia with poor weight gain. Before admission to the UMIT, the same management was followed as with patient case number 1, the blood glucose level at admission was 215 mg/dL. The results of the blood tests upon admission to the UMIT are presented in Table 1. As in case 1, the patient presented low C-peptide (0.05 ng/mL) and no pancreatic tissue.

Considering the records of the older brother, molecular analysis of the *PDX1* gene was also performed on this patient to look for specific molecular variants.

## Investigation

### Case number 1

The molecular analysis allowed the identification of two variants in the *PDX1* gene:

1. *PDX1* c.527G>A(p.Arg176Gln) in a state of heterozygosity classified as probably pathogenic, according to the PM1 (located at a mutational hotspot and/or in a well-established critical functional domain (e.g. active site of an enzyme) without benign variation); PM2 (absent from controls (or extremely infrequently if recessive)); PM5 (new missense change in an amino acid residue where a different missense change was determined to be seen as pathogenic); PP3 (multiple lines of computational evidence support a deleterious effect on the gene or gene product (conservation, evolutionary, splicing impact, etc.)) criteria of the American College of Medical Genetics and Genomics (ACMG), analyzed in 15 predictors that classify it as pathogenic.
2. *PDX1* c.571A>G(p.Lys191Glu), in a state of heterozygosity cataloged as a variant of uncertain significance, according to the PM1 (located at a mutational hotspot and/or in a critical and well-established functional domain (e.g. active site of an enzyme) without benign variation); PM2 (absent from controls (or extremely infrequently if recessive)); PP3 (multiple lines of computational evidence support a deleterious effect on the gene or gene product (conservation, evolutionary, splicing impact, etc.)) criteria of the ACMG, analyzed in 15 predictors, of which, 14 classify it as pathogenic and 1 with medium effect. This variant is not reported in the databases. The *PTF1A* gene did not report pathogenic variants.

### Case number 2

Molecular analysis allowed the identification of two variants in the *PDX1* gene:

1. *PDX1* c.527G>A(p.Arg176Gln) in a state of heterozygosity, classified as probably pathogenic, analyzed in 19 predictors that classify it as pathogenic.
2. *PDX1* c.571A>G(p.Lys191Glu), in a state of heterozygosity, cataloged as a variant of uncertain significance (VUS), analyzed* in silico*19 predictors which classify it as pathogenic.

## Treatment

### Case number 1

The patient was evaluated by the pediatric endocrinology service on suspicion for transient vs permanent neonatal diabetes. Initial management with continuous low-dose insulin infusion was considered (1–1.3 u/kg/day). Glucose values ranged between 30 and 342 mg/dL, so in 8 days, it was switched to fast insulin (Novorapid® 0.5 U/k/day), then slow insulin (Levemir® 0.5 U/k/day was added), and finally Glargine was administered.

To continue the study of the patient, pediatric gastroenterology was consulted. Within the extension studies, fecal elastase was requested, which reported less than 5.2 (μg/g) so replacement therapy with pancreatic enzymes was started (1300 IU before each intake and supplementation with fat-soluble vitamins).

The patient was hospitalized in the UMIT until he was 5 months old so that adequate metabolic control and good weight gain (64 g/day) was achieved; thereafter training for the mother was provided and it was possible to start complementary feeding for achieving a nutritional intake of 200 kcal/k/day with a hypercaloric formula for infants with an energy density of 1 kcal/1 mL. With this, better control of glucometry was achieved with an insulin pump. The patient was discharged at 5.5 months with a weight of 4.54 g, a height of 56 cm, an HC of 38.5 cm, and an improvement in glycosylated hemoglobin (HbA1C). The patient was discharged with an insulin infusion pump, pancreatic enzyme replacement therapy, intake of fat-soluble vitamins, and orders for outpatient control by complementary specialties. The final diagnosis was permanent neonatal diabetes and exocrine pancreatic insufficiency secondary to dysgenesis of the dorsal pancreas due to a mutation in the *PDX1* gene and secondary malnutrition.

### Case number 2

Taking into account the management records of case number 1, in this patient management with rapid insulin (Novorapid® 0.5 U/k/day) was indicated, later slow insulin (Levemir®0.5 U/k/day) was added. The same complementary tests were carried out in which the absence of pancreatic tissue, low C-peptide (0.05 ng/mL), and low fecal elastase were found (5 μg/g).

This patient was also in the UMIT until 5 months of age when adequate metabolic control was achieved with a therapeutic scheme similar to that of case number 1. Subsequently, with the adaptation of the insulin pump, enzyme replacement therapy pancreatic, and the contribution of fat-soluble vitamins, she left the clinic in good condition with a weight of 4355 g and a height of 58 cm.

The outflow diagnosis was similar to that of case number 1.

## Outcome and follow-up

Following hospital discharge, follow-up was determined by the multidisciplinary team every 3 months during the first year and subsequently, every 6 months to make the necessary adjustments to the insulin infusion pump. In June 2021, after follow-up by endocrinology, gastroenterology, and genetics, patient case number 1 was declared a candidate to receive growth hormone due to continued low weight and height. The HbA1C value at this follow-up was 7.9%. In this follow-up, the patient case number 2 was found with short stature, but the treatment with growth hormone has not yet been started because in Colombia treatment is only permitted after 4 years of age.

## Discussion

This article describes the first two cases in Colombia of PD secondary to a compound heterozygous mutation in the *PDX1* gene. In the case of the c.527G>A variant, it was possible to corroborate with a molecular study that this variant was inherited by the maternal line, which confirms the theory that the mother is a healthy carrier. As background, in 2015, a patient with a homozygous mutation in *PDX1* c.527G>A(p.Arg176Gln) whose main phenotype was diabetes was reported, without registering pancreatic agenesis (2). Pancreatic agenesis due to homozygous mutations in the *PDX1* gene was initially demonstrated in mice by Jonsson *et al.* (3) and later reported in humans by Stoffer *et al.* (4).

On the other hand, the *PDX1* c.571A>G(p.Lys191Glu) variant was confirmed as inherited from the paternal line, as it was identified in the father as a healthy carrier. Up to the date of writing this article, no other cases with these same variants have been reported in the literature.

The *PDX1* gene is located at 13q12.2, has a size of 6.32 Kb, is made up of two exons, and codes for a 283-amino acid homonymous protein that participates as a transcriptional activator of several genes, within the which are the genes for somatostatin, insulin, glucokinase, among others (5, 6). In addition, the PDX1 protein, which is detectable from the fifth week of gestation, is involved in the development of the pancreas as part of the protein complex that initiates a hierarchical sequence of events, allowing differentiation of the pancreas from pancreatic progenitor cells derived from the endodermal intestinal tube (7, 8).

As reported in the literature, the proportion of neonatal diabetes cases attributed to monogenic causes is around 50% (9); and about 3% of cases of permanent neonatal diabetes mellitus with permanent insulin requirement due to pancreatic agenesis are attributed to pathogenic variants of the *PDX1* gene, either in homozygosis, that is, the same variant in both alleles, or compound heterozygosis, understood as the presence of two different variants each in a gene locus (2), the latter being the one presented by the two brothers reported.

Due to the genetic heterogeneity that occurs in neonatal diabetes, it is important to recommend clinical evaluation by genetics, as well as the performance of molecular tests that allow establishing the etiology of the clinical picture (9). In terms of diagnosis, a new approach to the simultaneous analysis of several genes has recently been considered (10). According to the ISPAD guidelines (11), for the study of neonatal and monogenic diabetes, the use of panels is appropriate, although barriers to access to this type of study must still be overcome, such as the cost of the examination, at least in Latin America (12).

The study of fecal elastase in patients with neonatal diabetes and steatorrhea is also another valid way of approaching the diagnosis, since patients with exocrine pancreas involvement who suffer from steatorrhea may also have endocrine pancreas involvement. It can be the first step before requesting a genetic study in patients with these characteristics, as well as the total abdominal ultrasound to visualize the pancreas, which is considered the first-line and low-cost study (13).

Regarding insulin management, it is recognized that titration according to response should continue, since treatment should be personalized (14). In this sense, insulin management with a continuous infusion pump offers an alternative of flexibility and efficacy in dose adjustment (15). Regarding this therapy, in the last decade, it has been reported that continuous insulin infusion pump therapy generates better results than injection therapy in children under 5 years of age (16). Although some studies do not show a statistically significant improvement in glycosylated hemoglobin, clinically significant declines have been reported after 3 months of sustained therapy (17, 18, 19, 20). In the case of the patients presented, the total insulin dose ranged between 1 and 1.3 U/k/day. Although this dose is higher than that used in other studies, this can be attributed to the genetic findings found.

In conclusion, the multidisciplinary management that was carried out in these two cases led to a successful diagnosis and therapeutic approach.

## Declaration of interest

The authors declare that there is no conflict of interest that could be perceived as prejudicing the impartiality of the research reported.

## Funding

This research did not receive any specific grant from any funding agency in the public, commercial or not-for-profit sector.

## Patient consent

Written informed consent for publication of their clinical details and clinical images was obtained from the relatives of the patients.

## Author contribution statement

N Forero-Castro contributed to the definitive diagnosis, participated in the literature review, the writing process, and revisions of the manuscript. L C Ramírez, J C Celis and F A Silva Henao contributed to the definitive diagnosis and clinical management of the patients. F Leal-Valencia: contributed to the definitive diagnosis and clinical management of the patients, participated in the literature review, coordinated the manuscript writing process, and approved the final version.
